# Use of hypnotic drugs among Scandinavian children, adolescents, and young adults

**DOI:** 10.1192/j.eurpsy.2022.250

**Published:** 2022-09-01

**Authors:** R. Wesselhøft, L. Rasmussen, P. Jensen, P. Jennum, S. Skurtveit, I. Hartz, J. Reutfors, P. Damkier, M. Bliddal, A. Pottegård

**Affiliations:** 1 Mental Health Services in the Region of Southern Denmark, Child And Adolescent Psychiatry Odense, Odense C, Denmark; 2 Clinical Pharmacology, Pharmacy and Environmental Medicine, Institute Of Public Health, Odense C, Denmark; 3 Danish Center for Sleep Medicine, Rigshospitalet, Copenhagen University, Copenhagen N, Denmark; 4 Norwegian Institute of Public Health, Department Of Mental Disorders, Oslo, Norway; 5 Karolinska Institutet, Medicine Solna, Stockholm, Sweden; 6 Odense University Hospital, Dept. Of Clinical Biochemistry And Pharmacology, Odense C, Denmark

**Keywords:** pharmacoepidemiology, Child and adolescent, melatonin, drug utilisation

## Abstract

**Introduction:**

Hypnotic drug use in children and adolescents is widely debated.

**Objectives:**

To describe use of hypnotic drugs (melatonin, z-drugs and sedating antihistamines) among 5-24-year-old Scandinavians during 2012 to 2018.

**Methods:**

Aggregate-level data from public data sources in Sweden, Norway and Denmark. We calculated annual prevalence (users/1000 inhabitants) stratified by sex, age group and country. Quantity of use (Defined Daily Dose (DDD)/user/day) was estimated for Norway and Denmark.

**Results:**

Melatonin was most frequently used, with an increase from 2012 to 2018 in all countries. Sweden presented the highest rise (7 to 25/1,000) compared to Denmark (6 to 12/1,000) and Norway (10 to 20/1,000). The increase was strongest for females and 15-24-year-olds. Melatonin use was twice as common for males under age 15 years, and slightly more common for females thereafter. The annual prevalence of sedating antihistamine use doubled from 7 to 13/1,000 in Sweden, whereas it was more stable in Norway and Denmark, reaching 8/1,000 and 3/1,000, respectively. Z-drug use decreased in all countries, lowering to 4/1,000 in Sweden and Norway in 2018 and 2/1,000 in Denmark. The quantity of hypnotic use in Norway and Denmark was 1 DDD/user/day for melatonin, as compared to 0.1-0.3 for z-drugs and antihistamines.

**Conclusions:**

There is an increasing use of melatonin and sedating antihistamines among Scandinavian children, adolescents and young adults. The increase is more pronounced in Sweden compared to Norway and Denmark. This Scandinavian discrepancy could reflect variation in frequency of sleep problems or national variation in clinical practice or health care access.

**Disclosure:**

No significant relationships.

